# Sphaeroane and Neodolabellane Diterpenes from the Red Alga *Sphaerococcus coronopifolius*

**DOI:** 10.3390/md7020184

**Published:** 2009-05-19

**Authors:** Vangelis Smyrniotopoulos, Constantinos Vagias, Vassilios Roussis

**Affiliations:** Department of Pharmacognosy and Chemistry of Natural Products, School of Pharmacy, University of Athens, Panepistimiopolis Zografou, Athens 15771, Greece; E-Mails: esmiriniot@geol.uoa.gr; vagias@pharm.uoa.gr

**Keywords:** Sphaerococcus coronopifolius, diterpenes, neodolabellanes, sphaeroanes

## Abstract

Investigation of minor metabolites in the extracts of the red alga *Sphaerococcus coronopifolius* collected from the rocky coasts of Corfu Island in the Ionian Sea yielded two new diterpene alcohols, sphaerollanes I, and II (**1**, **2**) featuring neodolabellane skeletons, and the new sphaeroane diterpene alcohol 16-hydroxy-9S*-acetoxy-8-*epi*-isosphaerodiene-2 (**3**), along with two previously reported metabolites **4**, **5**. The structures of the new natural products, as well as their relative stereochemistry, were elucidated on the basis of extensive spectral analysis, including 2D-NMR experiments.

## 1. Introduction

All organisms biosynthesize secondary metabolites for certain essential physiological functions and exhibit complex chemical profiles, frequently including terpene metabolites. The numerous ways that the basic C_5_ units can be joined together and the different ecological pressures under which organisms have evolved justifies the enormous number and the diversity of the elaborated terpenoid structures [1].

*Sphaerococcus coronopifolius* has been the focus of previous chemical investigations, which showed the presence of interesting diterpenes with two, three and four ring carbon skeletons, most of which contain one or two bromine atoms [2,3].

**Figure 1:**
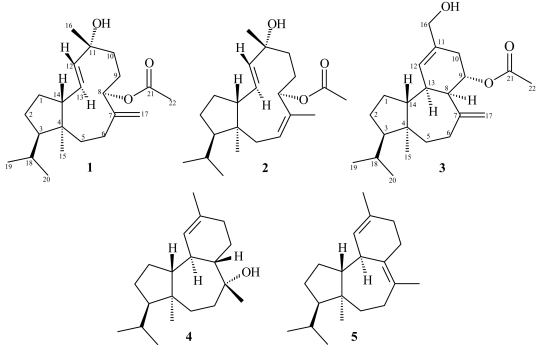
Structures of compounds **1**–**5**.

In the course of our ongoing investigations toward the isolation of bioactive metabolites from marine organisms of the Greek seas [4,5], we recently studied the chemical composition of the red alga *S. coronopifolius*, collected from the west coast of Corfu Island. In this report we describe the isolation and structure elucidation of two new diterpenes **1**, **2** with neodolabellane carbon skeletons, and one new sphaeroane diterpene **3**, along with the already described metabolites presphaerol (**4**) [6–8] and isosphaerodiene-1 (**5**) [8,9] (Figure 1). The structures of the new metabolites were elucidated by extensive spectroscopic analyses and their relative stereochemistry was established by NOESY experiments. Moreover, detailed analyses of the 1D- and 2D-NMR spectra allowed full assignment of the ^13^C- and ^1^H- data which had not been previously reported for **4** and **5**.

## 2. Results and Discussion

*S. coronopifolius* was collected in Palaiokastritsa Bay on the west side of Corfu Island and the CH_2_Cl_2_/MeOH extract of the freeze-dried alga was subjected to a series of column chromatography fractionations on silica gel, as well as normal, reversed and chiral phase high performance liquid chromatography (HPLC) separations to yield compounds **1** – **5** in pure form.

Sphaerollane I (**1**) was obtained as a colorless viscous oil. The molecular formula, C_22_H_36_O_3_, which was derived from HRMS and NMR data (Table 1), requires five degrees of unsaturation. The LREI-MS ions at *m/z* 330 [M-H_2_O]^+^, at *m/z* 288 [M-AcOH]^+^, and at *m/z* 270 [M-H_2_O-AcOH]^+^ indicated the presence of an acetate and a hydroxyl group. This was supported by the IR spectrum, which contained intense ester bands at 1,730 cm^−1^ (C=O stretch), 1,240 and 1,030 cm^−1^ (C–O stretch), and showed the presence of hydroxyl functionality (*v*_max_ 3,492 cm^−1^). Analysis of the NMR data (Table 1) revealed the presence of one acetate group (*δ*_H_ 2.06 s; *δ*_C_ 170.8 s, 21.2 q), a disubstituted double bond (*δ*_H_ 5.52 d and 5.66 dd; *δ*_C_ 137.3 d and 127.5 d), an olefinic methylene group (*δ*_H_ 4.74 br. t and 4.65 br. s; *δ*_C_ 149.8 s and 107.0 t), and a tertiary alcohol (*δ*_C_ 73.4 s). Interpretation of the ^1^H-^1^H COSY, ^1^H-^1^H TOCSY, HSQC, and HMBC data allowed the remaining two degrees of unsaturation to be assigned to a neodolabellane carbon skeleton. The H_3_-16 signal at *δ*_H_ 1.24 (s) showed HMBC correlations to C-10 (*δ*_C_ 38.2), C-11 (*δ*_C_ 73.4), and C-12 (*δ*_C_ 137.3), which placed the methyl on the quaternary oxygenated carbon adjacent to the 1,2-disubstituted double bond. Cross peaks in the COSY spectrum revealed couplings between H-10α (*δ*_H_ 1.66) and H-9b (*δ*_H_ 1.42), H-10β (*δ*_H_ 1.48) and H-9a (*δ*_H_ 1.89), and from both H_2_-9 to H-8 (*δ*_H_ 5.41). Furthermore, the H-8 methine signal showed HMBC correlations to C-9 (*δ*_C_ 28.3), C-10 (*δ*_C_ 38.2), C-7 (*δ*_C_ 149.8), C-17 (*δ*_C_ 107.0), and to the acetate signal at *δ*_C_ 170.8, confirming the position of methylene carbons C-9 and C-10 between the acetoxy-bearing carbon C-8 and the oxygenated carbon C-11. The correlations from H-5β (*δ*_H_ 1.28) and both H_2_-17 (*δ*_H_ 4.74 and 4.65) to C-6 (*δ*_C_ 28.4) required the presence of the exomethylene at C-7. The H_3_-15 signal at *δ*_H_ 0.77 (s) correlated with the C-3 (*δ*_C_ 55.4), C-4 (*δ*_C_ 47.1), C-5 (*δ*_C_ 37.0), and C-14 (*δ*_C_ 59.4) signals, allowing it to be placed at the C-4 bridge-head position. Homonuclear coupling between H-13 (*δ*_H_ 5.66) and H-14 (*δ*_H_ 2.18), and HMBC correlations of both H-1β (*δ*_H_ 1.47) and H-12 (*δ*_H_ 5.52) to C-14 (*δ*_C_ 59.4) completed the 11-membered ring by positioning the disubstituted double bond next to the ring junction. The correlations from both methyl signs H_3_-19 and H_3_-20 at *δ*_H_ 0.83 (d) and 0.95 (d), respectively, to C-3 (*δ*_C_ 55.4), C-18 (*δ*_C_ 30.3) clearly positioned the isopropyl group at C-3. The signal of H-1α at *δ*_H_ 1.36 (m), belonging to one of the remaining intercoupling methylene groups, showed HMBC correlation with carbon C-13 (*δ*_C_ 127.5). Thus, C-1 had to be connected to C-14, and the remaining C-2 methylene had to be connected to C-3, forming a cyclopentane ring.

**Figure 2:**
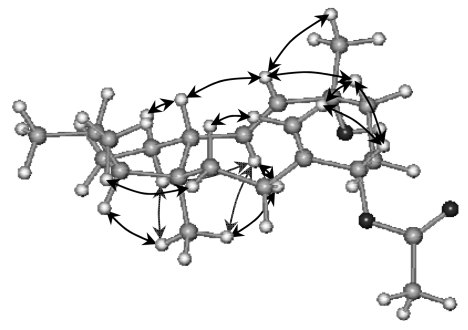
Relative configurations and key NOE correlations for compound **1**.

The relative stereochemistry shown for **1** (Figure 2) was assigned by interpretation of ^1^H-NMR coupling constants and NOESY data. The *E*-geometry of the 12,13-olefin was assigned on the basis of a 15.3 Hz coupling constant. The NOE correlations observed between H-1β and H-12 with H-14, as well as the correlations between H-1α, H-13 and H_3_-15, required a trans ring fusion and indicated that protons H-1β, H-12 and H-14 were on the same (upper) side of the compound, while H-1α, H-13 and H-15 were on the opposite. The stereochemistry at C-3 was deduced by the NOE correlation between proton H-3 and methyl protons H_3_-15. The H-8 signal showed a correlation to the H-10β signal, which in turn showed a NOE to the H-12 signal, thereby establishing the stereochemistry at C-8. The correlation between H-12 and H_3_-16 indicated that methyl H_3_-16 is also on the upper side of the ring, resulting in the (3R*,4S*,8S*,11R*,12*E*,14R*)-geometry proposed for sphaerollane I (**1**).

**Figure 3:**
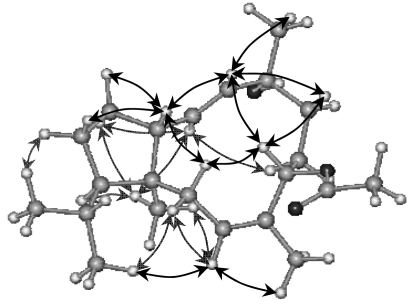
Relative configurations and key NOE correlations for compound **2**.

Compound **2** proved to have the same molecular formula as **1**, C_22_H_36_O_3_, which was derived from HRMS and NMR data (Table 1), and is an isomer of **1** that differs only in the location of one of the double bonds. The structural type and substitution pattern of **2** was elucidated by means of 1D- and 2D-NMR correlated spectroscopy including HSQC, HMBC and ^1^H-^1^H COSY. Sphaerollane II (**2**) contained a Δ^6^ trisubstituted double bond instead of the Δ^7,17^ olefinic methylene in **1**. In the ^1^H-NMR spectrum (Table 1), both H_2_-5 signals at *δ*_H_ 2.52 (dd) and 2.28 (ddq) were coupled to the H-6 olefinic signal at *δ*_H_ 5.25 (ddq) that along with methylene proton H-5α (*δ*_H_ 2.28) showed long-range couplings to the H_3_-17 methyl sign at *δ*_H_ 1.61 (t). The *Z* geometry of Δ^6^olefinic bond was assigned on the basis of NOE correlations between H-6 and H_3_-17, and between H-5β and H-8. Further analysis of the NOESY spectrum revealed that the relative stereochemistry at C-3, C-4, C-8, C-11 and C-14 was the same as in **1** (Table 1, Figure 3).

(3R*,4S*,8R*,9S*,13R*,14R*)-16-Hydroxy-9-acetoxy-8-*epi*-isosphaerodiene-2 (**3**) was isolated as a colorless oil. The high-resolution mass measurement established the molecular formula as C_22_H_34_O_3_, requiring six unsaturation equivalents. The IR spectrum showed bands that were assigned to ester (*v*_max_ 1,733, 1,240, 1,024 cm^−1^) and hydroxyl (*v*_max_ 3,367 cm^−1^) functionalities, respectively These assignments were supported by the positive LRCI-MS ions at *m/z* 329 [M+H-H_2_O]^+^, at *m/z* 287 [M+H-AcOH]^+^, at *m/z* 269 [M+H-AcOH-H_2_O]^+^, and at *m/z* 255 [M+H-AcOH-CH_2_OH]^+^ indicating the presence of an acetate and a primary hydroxyl group. The presence of five sp^2^ carbon atoms in the molecule, as deduced from the ^13^C-NMR and DEPT spectra (Table 2), being for one carbon-oxygen and two carbon-carbon double bonds, indicated compound **3** to be tricyclic. The ^1^H- and ^13^C-NMR spectra (Table 2) contained resonances for one acetate group (*δ*_H_ 1.99 s; *δ*_C_ 170.8 s, 21.2 q), a trisubstituted olefinic bond (*δ*_H_ 5.68 br. dd; *δ*_C_ 133.5 s and 127.4 d), an exomethylene (*δ*_H_ 4.79 br. s, 2H; *δ*_C_ 149.4 s and 114.1 t), and a primary alcohol (*δ*_H_ 3.99 br. s, 2H; *δ*_C_ 66.8 t). Extensive analyses of the 2D-NMR data of **3** including COSY, HSQC and HMBC spectra led to the unambiguous assignment of all protons and carbons on the sphaeroane skeleton. The H_2_-16 oxygenated methylene protons at *δ*_H_ 3.99 (br. s) showed heteronuclear long range couplings to C-10 (*δ*_C_ 32.7), C-11 (*δ*_C_ 133.5), and C-12 (*δ*_C_ 127.4), which confirmed the presence of the primary allylic alcohol at C-11. Correlations from H-8 methine (*δ*_H_ 2.74) and both H_2_-10 (*δ*_H_ 2.59 and 2.04) to C-9 (*δ*_C_ 68.7), as well as from H-9 (*δ*_H_ 5.35) to the carbonyl signal at *δ*_C_ 170.8 required the acetyl group to be positioned at C-9. The olefinic proton H-12 (*δ*_H_ 5.68) showed HMBC correlations to C-8 (*δ*_C_ 51.3) and C-13 (*δ*_C_ 41.6), thus positioning both the double bond and the acetate group next to the 6-membered ring junctions. The H_2_-17 olefinic methylene protons at *δ*_H_ 4.79 (br. s) correlated with C-6 (*δ*_C_ 30.8), C-7 (*δ*_C_ 149.4), and C-8 (*δ*_C_ 51.3), had to be placed on C-7. Long range heteronuclear couplings from H_2_-6 (*δ*_H_ 2.23 and 2.22) to both C-4 (*δ*_C_ 46.4) and C-5 (*δ*_C_ 38.9), and from H_2_-5 (*δ*_H_ 1.87 and 1.48) to C-7 (*δ*_C_ 149.4) were observed in the HMBC spectrum. The H_3_-15 methyl signal at *δ*_H_ 0.76 showed correlations to C-3 (*δ*_C_ 58.0), C-4 (*δ*_C_ 46.4), C-5 (*δ*_C_ 38.9), and C-14 (*δ*_C_ 53.7), and was placed on the quaternary carbon C-4. The cycloheptane ring was completed by the HMBC correlation observed between H-8 (*δ*_H_ 2.74) and C-14 (*δ*_C_ 53.7). The correlation of both H_3_-19 and H_3_-20 (*δ*_H_ 0.82 and 0.92) with C-3 (*δ*_C_ 58.0) and C-18 (*δ*_C_ 29.8), required the presence of the isopropyl group at C-3. The correlations observed between H-1α (*δ*_H_ 1.25) and C-13 (*δ*_C_ 41.6), and between H-2b (*δ*_H_ 1.28) and C-18 (*δ*_C_ 29.8), confirmed the place of methylene carbons C-1 and C-2 in the 5-membered ring.

**Figure 4:**
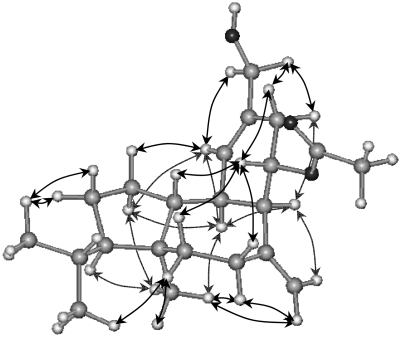
Relative configurations and key NOE correlations for compound **3**.

Stereochemical assignment of the chiral centers was facilitated by interpretation of ^1^H-NMR and NOESY spectra. NOE correlations between H_3_-15 and H-13 and H-1α required trans fusion between the 7-membered and the cyclopentane ring. Furthermore, the correlation between H_3_-15 and H-3 positioned both H-3 and H-13 co-facial to methyl H_3_-15. The strong NOEs observed between the pairs of protons H-9, H-14 and H-8, H-13 suggested the unusual for a sphaeroane skeleton cis-fusion of the 7-membered and the cyclohexene rings. Both C-8 and C-13 were assigned as R*, based on the NOE correlations of H_3_-15 with H_2_-17, of H_2_-17 with H-8, of the latter with H-10α, and of H_3_-15 with H-13, H-5α and H-6α. Moreover, the NOEs observed between H-14 and H-9, and between H-9 and H-5β, H-6β and H-10β, secured the relative stereochemistry at chiral centers C-9 and C-14, thereby defining the structure as (3R*,4S*,8R*,9S*,13R*,14R*)-16-hydroxy-9-acetoxy-8-*epi*-isosphaerodiene-2 (**3**) (Figure 4).

**Scheme 1:**
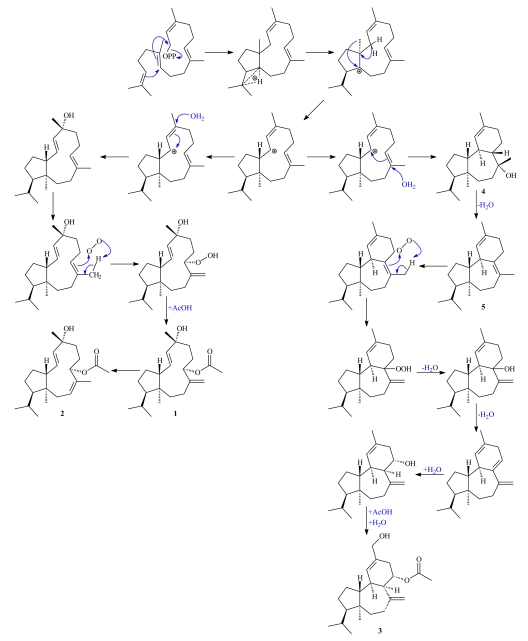
Proposed biogenetic origin of **1**–**5**.

Along with the above described metabolites two previously reported compounds, presphaerol (**4**) [6–8] and isosphaerodiene-1 (**5**) [8, 9], were isolated and spectroscopically characterized. Complete ^1^H- and ^13^C-NMR assignments, based on extensive analyses of their 2D-NMR spectra, are now provided for the first time for **4** and **5**. The co-occurrence of compounds **1**, **2** and **3** with the previously described **4** and **5** in the same organism indicated the possibility that they all derive from a common precursor neodolabellane cation [10], as illustrated in the proposed biogenetic pathway (Scheme 1). Thus, successive nucleophilic substitution of a H_2_O molecule, oxidation of the trisubstituted olefinic bond [11], and acetylation can yield the neodolabellanes **1** and **2**. Alternatively, nucleophilic attack of a H_2_O molecule on the trisubstituted Δ^7^ double bond can produce presphaerol (**4**) [10], which through water elimination generates isosphaerodiene-1 (**5**). Metabolite **3** can be derived from **5** by oxidation of Δ^7^ double bond and successive eliminations, additions of H_2_O molecules, and a final acetylation.

Diterpenes of the neodolabellane class have previously been isolated only from soft corals of the genera *Cespitularia*, *Clavularia*, and *Lobophytum* of the Indian and Pacific Ocean [12–16], while diterpenes of the sphaeroane skeleton have been isolated, only from the red alga *S. coronopifolius* and the macromycete *Mycena tintinnabulum* [17].

## 3. Experimental Section

### 3.1. General Experimental Procedures

Optical rotations were measured on a Perkin-Elmer model 341 polarimeter with a 10 cm cell. UV spectra were acquired in spectroscopic grade CHCl_3_ on a Shimadzu UV-160A spectrophotometer. IR spectra were obtained using a Paragon 500 Perkin-Elmer spectrophotometer. NMR spectra were recorded using a Bruker AC 200 and Bruker DRX 400 spectrometers. Chemical shifts are given on a *δ* (ppm) scale using TMS as internal standard. The 2D-NMR experiments (^1^H-^1^H COSY, HSQC, HMBC, NOESY) were performed using standard Bruker microprograms. The structures in Figures 2, 3 and 4 were generated and optimised (energy: 43.50, 38.24 and 37.77 Kcal/mole, respectively) by “HyperChem^™^ 7.0” molecular modelling and simulation software (force field: MM+; optimisation algorithm: Polak-Ribiere). High-Resolution mass spectral data were provided by the University of Notre Dame, Department of Chemistry and Biochemistry, Indiana, USA. Low-Resolution Electron Impact or Chemical Ionisation MS data were recorded on a Thermo DSQ Mass Detector using Direct Exposure Probe (DEP) and methane as the CI gas. Vacuum Liquid Chromatography (VLC) separation was performed with Kieselgel 60 (Merck), gravity column chromatography (GCC) was performed with Kieselgel 60H (Merck), thin layer chromatography (TLC) was performed with Kieselgel 60 F_254_ aluminum support plates (Merck) and spots were detected after spraying with 15% H_2_SO_4_ in MeOH and charring. HPLC separations were conducted using an Agilent 1100 system equipped with refractive index detector and a SupelcoSil 5μ (250×10 mm) HPLC normal phase column or a Kromasil 100 C_18_ 5μ (250×8 mm) HPLC reversed phase column or a Chiralcel OD 5μ (250×4.6 mm) analytical chiral column.

### 3.2. Plant Material

*S. coronopifolius* was collected by SCUBA diving in Palaiokastritsa Bay at the west coast of Corfu island, Greece, at a depth of 10–15 m in May of 2002. A specimen is kept at the Herbarium of the Laboratory of Pharmacognosy and Chemistry of Natural Products, University of Athens (ATPH/MO/201).

### 3.3. Extraction and Isolation

*S. coronopifolius* was initially freeze-dried (291.4 g dry weight) and then exhaustively extracted with mixtures of CH_2_Cl_2_/MeOH (3/1) at room temperature. The combined extracts were concentrated to give a dark green residue (8.20 g), which was later subjected to vacuum liquid chromatography on silica gel, using a 10% step gradient of cyclohexane-EtOAc elution sequence. Fraction F2 (4.01 g), eluted with 20% EtOAc in cyclohexane, was fractionated by gravity column chromatography, using a 2% step gradient of cyclohexane-EtOAc. Fraction F2.8 (524.5 mg), eluted with 8% EtOAc in cyclohexane, was further separated by silica gel GCC using isocratic 5% EtOAc in cyclohexane. Fraction F2.8.15 (53.3 mg) was subjected repeatedly to reverse phase HPLC using MeOH as mobile phase to afford pure **4** (1.9 mg) and **5** (1.8 mg). The CH_3_CN soluble portion (173.4 mg) of fraction F2.11 (50% EtOAc in cyclohexane) (199.6 mg) was subjected to reverse phase HPLC, using CH_3_CN as mobile phase. Peak F2.11.7 (retention time 17.9 min) (12.4 mg) was further purified by normal phase HPLC with 20% *n*-hexane in CHCl_3_. Compounds **1** (4.9 mg) and **2** (0.9 mg) were isolated by chiral HPLC with 0.5% *i*-PrOH in *n*-hexane as eluent, from peak F2.11.7.2 (retention time 9.0 min) (5.8 mg). The CH_3_CN soluble portion (323.4 mg) of fraction F5 (70% EtOAc in cyclohexane) (446.6 mg) was subjected to reverse phase HPLC, using CH_3_CN as mobile phase. Peak F5.12 (retention time 11.6 min) (51.6 mg) was further separated by normal HPLC with 30% *n*-hexane in CHCl_3_ to yield pure **3** (4.4 mg).

#### Sphaerollane-I (**1**)

Colorless oil; [α]_D_ +81.8 (c 2.90, CHCl_3_); UV (CHCl_3_) λ_max_ (log *ɛ*) 255 (0.72) nm; IR (CHCl_3_) ν_max_ 3,492, 1,730, 1,240, 1,030, 970, 896 cm^−1^; NMR data (CDCl_3_), see Table 1; EIMS 70 eV, m/z (rel. int. %) 330 [M-H_2_O]^+^ (3), 306 [M-CH_2_CO]^+^ (29), 288 [M-AcOH]^+^ (37), 270 [M-AcOH-H_2_O]^+^ (44), 255 [M-AcOH-H_2_O-Me]^+^ (20), 245 [M-AcOH-*i*Pr]^+^ (72), 227 [M-AcOH-H_2_O-*i*Pr]^+^ (71), 199 (17), 171 (31), 133 (45), 91 (69), 43 [CH_3_CO]^+^ (100); HRFAB-MS (m/z): 349.2732 [M+H]^+^ (calcd for C_22_H_37_O_3_, 349.2743).

#### Sphaerollane-II (**2**)

Colorless oil; [α]_D_ +5.5 (c 1.10, CHCl_3_); UV (CHCl_3_) λ_max_ (log *ɛ*) 250 (1.76) nm; IR (CHCl_3_) ν_max_ 3,422, 1,728, 1,240, 1,014, 973 cm^−1^; NMR data (CDCl_3_), see Table 1; CIMS m/z (rel. int. %) 331 [M+H-H_2_O]^+^ (1), 289 [M+H-AcOH]^+^ (11), 271 [M+H-AcOH-H_2_O]^+^ (38), 255 [M+H-AcOH-H_2_O-Me]^+^ (9), 245 [M+H-AcOH-*i*PrH]^+^ (14), 227 [M+H-AcOH-H_2_O-*i*PrH]^+^ (8), 173 (14), 147 (9), 119 (11), 105 (8), 60 [AcOH]^+^ (100); HRFAB-MS (m/z): 348.2674 [M]^+^ (calcd for C_22_H_36_O_3_, 348.2664).

#### 16-Hydroxy-9S*-acetoxy-8-epi-sphaerodiene-2 (**3**)

Colorless oil; [α]_D_ +10.3 (c 2.90, CHCl_3_); UV (CHCl_3_) λ_max_ (log *ɛ*) 244 (1.95) nm; IR (CHCl_3_) ν_max_ 3,367, 1,733, 1,240, 1,024, 888 cm^−1^; NMR data (CDCl_3_), see Table 2; CIMS m/z (rel. int. %) 347 [M+H]^+^ (1), 329 [M+H-H_2_O]^+^ (9), 287 [M+H-AcOH]^+^ (29), 269 [M+H-AcOH-H_2_O]^+^ (100), 255 [M+H-AcOH-CH_2_OH]^+^ (11), 243 [M+H-AcOH-*i*PrH]^+^ (8), 225 [M+H-AcOH-H_2_O-*i*PrH]^+^ (12), 213 [M+H-AcOH-CH_2_OH-*i*Pr]^+^ (7), 171 (14), 145 (13), 137 (16), 105 (13), 60 [AcOH]^+^ (38); HRFAB-MS (m/z): 347.2586 [M+H]^+^ (calcd for C_22_H_35_O_3_, 347.2589).

## Figures and Tables

**Table 1:** NMR data^a^ of compounds **1** – **2**.

	1					2				

Pos.	*δ*_H_	mult, *J*	NOESY	*δ*_C_^b^	HMBC (C→H)	*δ*_H_	mult, *J*	NOESY	*δ*_C_^b^	HMBC (C→H)

1	β 1.47 α 1.36	m m	14 13, 15	27.9 t	2, 3	β 1.57 α 1.41	m m	14 13, 15	28.2 t	2β, 3
2	a 1.85 b 1.81	m m		27.1 t	1α, 1β, 18	α 1.87 β 1.30	m m	20 14	28.1 t	3
3	1.21	m	15	55.4 d	1α, 1β, 2a, 15, 18, 19, 20	1.29	m	15	57.0 d	15, 19, 20
4				47.1 s	1α, 2a, 2b, 3, 5β, 15				47.1 s	14, 15
5	α 2.07 β 1.28	m m	20 17b	37.0 t	3, 15	β 2.52 α 2.28	dd 14.9, 12.0 ddq 14.9, 6.2, 1.2	8, 14 6, 19	36.3 t	14, 15
6	α 2.16 β 1.97	m m	13, 15	28.4 t	5β, 17a, 17b	5.25	ddq 12.0, 6.2, 1.2	5α, 15, 17, 19	127.1 d	5α, 5β, 8, 17
7				149.8 s	5β, 8, 17a				132.8 s	5α, 17
8	5.41	brd 7.9	10β, 17a	75.7 d	6β, 9b, 10α,10β, 17a, 17b	5.86	dd 9.1, 1.6	5β, 12, 9α, 10β	73.8 d	9α, 17, 10α, 10β
9	a 1.89 b 1.42	m m		28.3 t	8, 10α, 10β	β 1.76 α 1.51	m m	8, 13	27.5 t	8, 10α
10	α 1.66 β 1.48	ddd 14.5, 7.9, 1.2 m	8, 12, 17a	38.2 t	8, 9a, 9b, 16	α 1.63 β 1.49	m m	8, 12	38.3 t	8, 9α, 16
11				73.4 s	9a, 9b, 10α, 12, 13, 16				73.0 s	12, 13, 16
12	5.52	d 15.3	10β, 14, 16	137.3 d	10α, 13, 16	5.66	d 15.3	8, 10β, 14, 16	137.1 d	10α, 13, 16
13	5.66	dd 15.3, 10.0	1α, 6α, 15	127.5 d	1α, 12	5.49	dd 15.3, 10.4	1α, 9α, 15	129.1 d	12
14	2.18	m	1β, 12	59.4 d	3, 5β, 15	2.22	m	1β, 2β, 5β, 12	59.7 d	5β, 12, 15
15	0.77	s	1α, 3, 6α, 13	10.9 q	3, 5β	0.67	s	1α, 3, 6, 13	12.5 q	14
16	1.24	s	12	30.7 q	10α	1.29	s	12	30.3 q	
17	a 4.74 b 4.65	brt 1.2 brs	8, 10β 5β	107.0 t	6β, 8	1.61	t 1.2	6	17.6 q	6, 8
18	1.56	brhept 6.6		30.3 d	3, 19, 20	1.53	m		31.0 d	19, 20
19	0.83	d 6.6		22.5 q	3, 18, 20	0.98	d 6.6	5α, 6	22.6 q	20
20	0.95	d 6.6	5α	23.5 q	3, 18, 19	0.85	d 6.6	2α	22.8 q	19
21				170.8 s	8, 22				171.3 s	8, 22
22	2.06	s		21.2 q		1.99	s		21.3 q	

**Table 2:** NMR data of compounds **3** – **5**.

	3^a^					4^b^			5^b^		

Pos.	*δ*_H_	mult, *J*	NOESY	*δ*_C_^c^	HMBC (C→H)	*δ*_H_	mult, *J*	*δ*_C_^c^	*δ*_H_	mult, *J*	*δ*_C_^c^

1	β 1.72 α 1.25	m m	12 12, 13, 15	27.8 t	14	a 1.69 b 1.38	m m	27.2 t	a 1.34 b 1.09	m m	26.4 t

2	a 1.72 b 1.28	m m	19 19	26.0 t	3	a 1.66 b 1.16	m m	28.2 t	a 1.65 b 1.35	m m	25.9 t

3	1.15	m	15	58.0 d	1α, 2b, 5β, 15, 19, 20	1.06	m	59.4 d	0.95	m	58.7 d

4				46.4 s	1β, 2a, 3, 5α, 5β, 6α, 6β, 15			45.3 s			46.4 s
5	α 1.87 β 1.48	ddd 13.7, 7.0, 5.8 m	15, 20 9	38.9 t	6α, 6β, 14, 15	a 1.50 b 1.49	m m	37.8 t	a 1.85 b 1.06	dd 12.8, 7.4 t 12.8	40.0 t
6	α 2.23 β 2.22	m m	15, 17 9	30.8 t	5α, 5β, 17	a 1.73 b 1.36	m m	37.2 t	a 2.46 b 1.72	m m	30.3 t
7				149.4 s	5α, 5β, 6α, 6β, 17			74.4 s			129.0 s
8	2.74	dd 11.2, 5.4	10α, 13, 17	51.3 d	6α, 6β, 9, 10β, 12, 17	1.67	m	48.2 d			135.8 s
9	5.35	ddd 11.2, 8.3, 6.2	5β, 6β, 10β, 14	68.7 d	8, 10α, 10β	a 1.74 b 1.49	m m	22.5 t	a 1.59 b 1.11	ddd 13.7,4.2, 3.7 dt 13.7, 7.9	26.0 t
10	β 2.59 α 2.04	dd 17.0, 6.2 dd 17.0, 8.3	9, 16 8, 16	32.7 t	8, 12, 16	a 1.91 b 1.89	m m	30.8 t	a 1.94 b 1.33	m m	30.2 t
11				133.5 s	10α, 10β, 16			132.4 s			133.9 s
12	5.68	brdd 2.9, 1.2	1α, 1β, 13, 16	127.4 d	10α, 10β, 13, 16	5.39	dm 3.5	126.8 d	5.42	brs	124.6 d
13	2.28	m	1α, 8, 12, 15	41.6 d	1α, 8, 9, 12, 17	2.45	dm 3.5	36.1 d	2.99	brs	37.3 d
14	1.65	m	9	53.7 d	5α, 8, 15	1.61	m	50.4 d	1.35	m	53.0 d
15	0.76	s	1α, 3, 5α, 6α, 13, 17	13.9 q	3, 5β	0.90	s	15.1 q	0.77	s	13.0 q
16	a 3.99 b 3.99	brs brs	10α, 10β, 12	66.8 t	10β, 12	1.67	brs	23.7 q	1.73	brs	24.1 q
17	a 4.79 b 4.79	brs brs	6α, 8, 15	114.1 t	6α, 6β, 8	1.10	s	29.6 q	1.76	brs	20.7 q
18	1.53	m		29.8 d	2b, 3, 19, 20	1.51	m	30.9 d	1.49	dhept 9.1, 6.6	30.9 d
19	0.82	d 6.6	2a, 2b	22.4 q	18, 20	0.89	d 6.7	22.9 q	0.96	d 6.6	23.5 q
20	0.92	d 6.6	5α	23.7 q	18, 20	0.98	d 6.7	23.6 q	0.86	d 6.6	23.2 q
21				170.8 s	9, 22						
22	1.99	s		21.2 q							
